# Age-related changes in the impact of valence on self-referential processing in female adolescents and young adults

**DOI:** 10.1016/j.cogdev.2021.101128

**Published:** 2022

**Authors:** M.E. Moses-Payne, G. Chierchia, S.-J. Blakemore

**Affiliations:** a UCL Institute of Cognitive Neuroscience, London WC1N 3AZ, UK; b University of Cambridge, Department of Psychology, Downing Street, Cambridge CB2 3EB, UK

**Keywords:** Adolescence, Self-reference, Memory, Self-concept

## Abstract

Adolescence is a period of self-concept development. In the current study, females aged 11–30 years (N = 210) completed two self-referential tasks. In a memory task, participants judged the descriptiveness of words for themselves or a familiar other and their recognition of these words was subsequently measured. In an associative-matching task, participants associated neutral shapes to either themselves or a familiar other and the accuracy of their matching judgements was measured. In the evaluative memory task, participants were more likely to remember self-judged than other-judged words and there was an age-related decrease in the size of this self-reference effect. Negative self-judgements showed a quadratic association with age, peaking around age 19. Participants were more likely to remember positive than negative words and there was an age-related increase in the magnitude of this positivity bias. In the neutral shapes task, there were no age-related changes in the self-reference effect. Overall, adolescent girls showed enhanced processing of self-relevant stimuli when it could be used to inform their self-concept and especially when it was negative.

## Highlights

• Negative self-judgements showed a quadratic association with age, peaking around age 19.
• Participants correctly recognized more self-judged than other-judged words and more positive than negative words.
• The magnitude of the self-reference effect, remembering more self- than other-judged words, showed an age-related decrease.
• The magnitude of the positivity bias, remembering more positive than negative words, showed an age-related increase.
• When stimuli were neutral shapes rather than evaluative words, there was no age-related change in the self-reference effect.

Adolescence begins with the onset of puberty and ends with the assumption of a stable, adult role (Blakemore and Mills, 2014, Patton et al., 2016). By this definition, adolescence is a period in which individuals construct an autonomous sense of self (Koepke & Denissen, 2012). It has been proposed that, compared with other ages, adolescents are more aware of and concerned with their self-concept (Elkind & Bowen, 1979, Sebastian, Burnett, & Blakemore, 2008, Brown, 2013, Erikson, 1968). Here, we assessed whether self-concept construction during adolescence would be reflected in heightened self-referential processing.

To assess self-referential processing, we evaluated the ‘self-reference effect’ (SRE), whereby stimuli associated with the self are better perceived or remembered than stimuli associated with others. For example, participants correctly recognise more words that were previously judged as descriptive of the self, compared with words that were judged as descriptive of another person (e.g. Harry Potter) or for their semantic or perceptual features (Leshikar, Dulas, & Duarte, 2015, Rogers, Kuiper, & Kirker, 1977). In associative-matching paradigms, participants show an SRE even when simply instructed to associate one shape stimuli with themselves and another shape stimuli with another person (Sui, He, & Humphreys, 2012). In such a paradigm, participants are generally faster and more accurate at processing self-associated shapes compared with other-associated shapes (Sui, He, & Humphreys, 2012).

The SRE has been demonstrated in children as young as 5 years (Sui & Zhu, 2005), in adolescents (Dégeilh et al., 2015) and in adults (Conway, 2005, Johnson et al., 2002J, Leshikar, Dulas, & Duarte, 2015, Rogers, 1977, Rogers, Kuiper, & Kirker, 1977, Sui, He, & Humphreys, 2012, Sui & Humphreys, 2015, Symons & Johnson, 1997). Previous work has suggested that the SRE shows an age-related increase (increased difference in recall memory between self- and other-judged words) from age 7–11 (Halpin, Puff, Mason, & Marston, 1984, Hammen & Zupan, 1984, Ray et al., 2009). However, little is known about the trajectory of the SRE between early adolescence and adulthood. Here, we hypothesised that younger adolescent girls would find self-relevant stimuli particularly salient as they seek to construct their self-concept, and therefore show a larger SRE than in adulthood when the self-concept becomes more certain.

Self-referential recognition memory paradigms employ word stimuli that are valenced (as opposed to associative-matching tasks that employ neutral shapes). This allows for a separate line of analysis on the impact of valence on self-referential processing. Valence may be a particularly important factor in adolescent self-referential processing, as previous evidence has suggested that adolescents may be more willing to endorse negative statements about themselves (van der Aar, Peters, & Crone, 2018). Indeed, negative self-judgements appear to increase during adolescence, peaking around ages 15–17 years, and then decrease in early adulthood (McArthur et al., 2019, van der Cruijsen et al., 2018).

Assessing memory might give new insights into the effects of valence on self-referential processing compared with simply asking participants to self-report the descriptiveness of words. Previous evidence suggests that adolescents show a negativity bias, recalling more negative than positive words (Bone, Lewis, Roiser, Blakemore, & Lewis, 2021) compared with children, who recall equal numbers of positive and negative words (Quas *et al.* 2016). However, this depends on the conditions under which stimuli are encoded and retrieved, for example, whether the study employs a free recall or recognition memory test (Neshat-Doost, Taghavi, Moradi, Yule, & Dalgleish, 1998). In adults, some studies report a similar memory advantage for both positive or negative words compared with neutral words (in recognition memory: Adelman & Estes, 2013) whereas others report a memory advantage exclusively for negative (in recognition memory: Inaba, Nomura, & Ohira, 2005; Santaniello et al., 2018) or even positive words (in free recall: Herbert, Junghofer, & Kissler, 2008; and recognition memory: Lee & Potter, 2020). Less is known about valence effects on memory performance when stimuli are encoded in a self-referential condition and there has been little research addressing age-related change in self-referential memory valence effects (Kauschke, Bahn, Vesker, & Schwarzer, 2019).

Adolescence is associated with heightened self-consciousness and propensity to social anxiety (Beesdo, Knappe, & Pine, 2009, Beesdo-Baum & Knappe, 2012, Caouette and Guyer, 2014, Elkind & Bowen, 1979, Rankin, Lane, Gibbons, & Gerrard, 2004, Somerville et al., 2013). Self-consciousness refers to the disposition to attend to the self, self-focussed attention or an awareness of self-referent information (Fenigstein, Scheier, & Buss, 1975, Mor et al., 2010, Wu & Watkins, 2006). Therefore, if the self-reference effect reflects attention to self-relevant stimuli, we would expect this to be associated with heightened self-consciousness. Social anxiety typically refers to a sensitivity to negative evaluations from others (Beesdo, Knappe, & Pine, 2009). Individuals use evaluations from others to inform their sense of self (Gallagher, 2000). Adolescents have been shown to be particularly sensitive to others’ evaluations of them (Brown, 2013), and may be more likely than other ages to integrate others’ views into their own self-concept (Pfeifer *et al.* 2009). Here, we sought to investigate how self-consciousness and social anxiety influence the development of adolescents’ self-referential processing as they emerge into adulthood.

Typically, adolescent girls self-report heightened self-consciousness (Rankin, Lane, Gibbons, & Gerrard, 2004, Rosenberg & Simmons, 1975), greater propensity for social anxiety (Beesdo, Knappe, & Pine, 2009) and lower self-esteem (Quatman & Watson, 2001) than do adolescent boys. However, when measuring self-concept development (via competency ratings, trait descriptor ratings or in self-reference memory tasks), gender differences are not consistently reported and seem to depend on domain. For example, previous work has shown that boys’ beliefs about their maths and language or arts competencies decreased more rapidly than girls’ between ages 6 and 18 years (Jacobs, Lanza, Osgood, Eccles, & Wigfield, 2002). This was not the case for sports, where boys maintained higher competency beliefs than girls over time (Jacobs, Lanza, Osgood, Eccles, & Wigfield, 2002). More recently, McArthur et al. (2019) reported no gender difference in the trajectory of positive and negative self-schema (proportion of words both judged as descriptive and subsequently remembered, out of all positive or negative words judged) across age but showed that at age 13, on average, girls reported higher levels of positive self-schema than boys. However, in many previous studies using self-judgements, gender differences in self-referential processing were not investigated (Pfeifer et al., 2009, Pfeifer et al., 2013, Pfeifer, Lieberman, & Dapretto, 2007, van der Aar et al., 2018, van der Cruijsen et al., 2018) and most previous research studied US-based samples. Here, we recruited only female participants because of their heightened self-consciousness and propensity to social anxiety (Beesdo, Knappe, & Pine, 2009, Rankin, Lane, Gibbons, & Gerrard, 2004, Rosenberg & Simmons, 1975).

Overall, we aimed to assess how female adolescents and young adults might build upon their self-concept by prioritising self-relevant information (the self-reference effect). To do this, we measured self-referential processing, and the impact of valence on self-referential processing, in female participants aged 11–30. We employed two tasks: a recognition memory (words) task and an associative-matching (shapes) task, to contrast self-referential processing of stimuli that are evaluative (words task) versus neutral (shapes task). We measured self-consciousness and social anxiety using a self-report scale (Takishima-Lacasa, Higa-McMillan, Ebesutani, Smith, & Chorpita, 2014) in order to assess the relationship between these measures and self-referential processing.

We hypothesised that the youngest participants would be most motivated to develop their self-concept and would find self-relevant stimuli most salient. Therefore, we predicted that the youngest participants would show the largest SRE in both the evaluative and neutral tasks (i.e. a larger difference in recognition of self-judged compared with other-judged words and a larger difference in speed and accuracy of judgements for self-associated shapes compared with other-associated shapes). We predicted that the SRE would show an age-related decrease as participants emerge into adulthood and their self-concept becomes more stable (Prediction 1). Based on previous literature, we predicted that negative self-judgements would increase across the adolescent years and decrease into adulthood (Prediction 2) and that memory for self-judged negative words would follow the same pattern with age (Prediction 3). We also investigated whether heightened concern with others’ opinions (measured by self-report public self-consciousness and social anxiety) would be associated with increased negative self-judgements (Prediction 4) and whether heightened self-reflectiveness (measured by self-report private self-consciousness) would be associated with an increased self-reference effect (Prediction 5).

## 1 Methods

### 1.1 Participants and recruitment

**Fig. 1:**
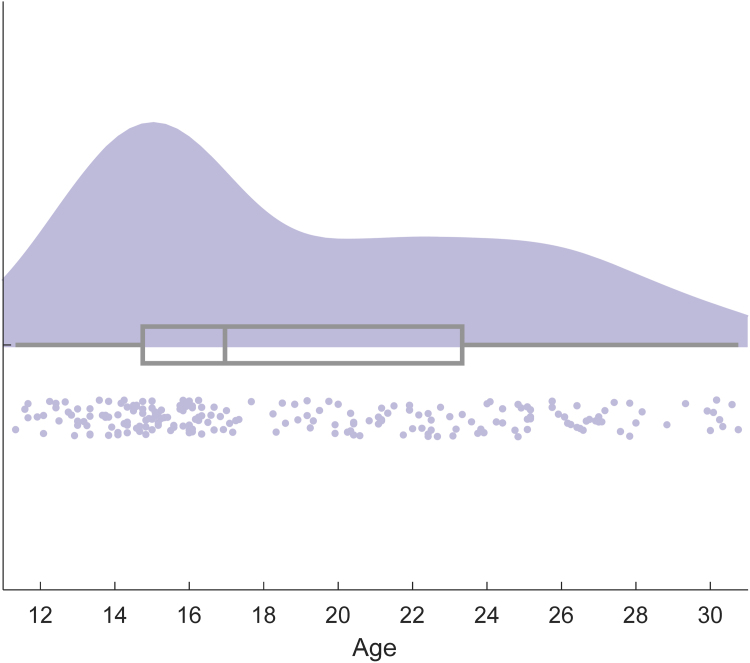
Distribution of sample across age. *Note.* Participants were aged 11.33–30.75. Box and whisker plot in grey shows min and max (ends of whiskers), lower quartile and upper quartile (ends of box) and median (line through box) of age. Dots represent individual participants and shaded area represents probability density.

We recruited 210 female participants aged 11.33–30.75 years (mean: 19.17, SD: 5.29; see Fig. 1). The lower age bound was chosen in order to capture the start of adolescence. The upper age bound was chosen to capture the end of adolescence (defined as mid-20s; Sawyer, Azzopardi, Wickremarathne, & Patton, 2018) and emerging adulthood. We expected that the development of most cognitive processes would have stabilised by age 30 (Becht et al., 2021, Craik & Bialystok, 2006, Mills et al., 2016). Females only were recruited so that gender differences would not have to be accounted for and because of their reported heightened self-consciousness and vulnerability to social anxiety (Beesdo, Knappe, & Pine, 2009, Rankin, Lane, Gibbons, & Gerrard, 2004). Participants were recruited from local secondary schools or university subject databases and paid £10 for their participation. Parents provided informed consent for participants under the age of 18. Participants 18 and over provided informed consent themselves. Individuals with diagnosed developmental conditions, including dyslexia, dyscalculia and autism spectrum conditions were excluded from the study. All participants reported normal or corrected-to-normal vision. The study was approved by UCL Research Ethics Committee (Project ID: 3453/001).

Sixty-seven participants (~32%) were White-British, 33 (~16%) were Indian, 25 (~12%) were Chinese, 23 (~11%) were White-Other, 22 (~10%) were Asian-Other, 21 (~10%) were mixed, 13 (~6%) were Black-British or Black-African, four (~2%) were Bangladeshi and two (~1%) reported other ethnicities. All participants were fluent in English. One hundred and fifty-eight participants reported English as their first language, with three participants listing English alongside another language and 49 participants reported a first language other than English.

### 1.2 Self-referential processing tasks

Participants completed a recognition memory (words) task and an associative-matching (shapes) task. In both tasks, participants were asked about stimuli that referred to the participants themselves or another person. Participants chose a female ‘familiar other’ whom they did not know personally (e.g. celebrity or fictional character). Once chosen, participants were asked to make a one-off rating of their familiarity with the chosen other on a scale from 0 “Not familiar at all” to 10 “Very familiar”, and their likeability from 0 “Don’t like her at all” to 10 “Like her very much”. This was done in order to ensure that participants across age chose to judge words in relation to people they were similarly familiar with and found similarly likeable, and could be used as a covariate in the case that age-related differences were found.

#### 1.2.1 Recognition memory (words) task

The words task consisted of two stages – encoding and retrieval.

##### 1.2.1.1 Encoding

During encoding, participants were asked to judge how well different words described themselves (‘Does this word describe you?’) or their chosen familiar other (‘Does this word describe [e.g. Hermione Granger]?’). Participants viewed the cue for 1000 ms followed by the person-descriptive word for 1000 ms. Participants judged the descriptiveness of the word on a 11-point scale from 0 (‘Does not describe me/her at all’) to 10 (‘Totally describes me/her’). The slider handle remained hidden until the participant’s first click (to avoid anchoring effects) and participants were given 7000 ms to make their rating.

Words were selected from Anderson (1968) and Kirby & Gardner, 1972 and were judged, prior to the study, by two adolescents aged 11 and 13 to ensure the youngest participants in the study would be able to comprehend the words. Post-hoc checks using age of acquisition data (Brysbaert & Biemiller, 2017 data available for 108/128 words) showed that the mean age of acquisition for the word stimuli was 4.32 years old and none of the words had an age of acquisition later than 10 years old (see Table S2).

Participants judged a total of 64 words based on how well they described themselves (32 words: 18 positive, 14 negative) or their chosen familiar other (32 words: 18 positive, 14 negative). Words were coded as positive or negative using a median split on the likeability or social desirability ratings (taken from Anderson, 1968 and Kirby & Gardner, 1972 respectively; see Table S2 for ratings).

##### 1.2.1.2 Retrieval

During retrieval, participants were given a surprise recognition test for words presented in the encoding stage. Participants were tested on all 64 target words, seen in the encoding stage, alongside 64 distractor words (with the same ratio of positive and negative words). Thus, participants made recognition judgements for 128 words in total. Participants viewed the recognition question (‘Have you seen this word before?’) for 1000 ms followed by the word for 1000 ms and pressed the left or right arrow to indicate their response. Recognition judgements were self-paced, both the question and word remained on the screen during the judgement.

##### 1.2.1.3 Allocation of words

The allocation of words to either target or distractor was pseudo-randomised across participants. To this end, the 128 words were split into eight lists of 16 words (see Table 1, each column corresponds to a list of words). For each participant, an algorithm selected at random four lists to be displayed during the encoding stage (two lists for self-judgements, two lists for other-judgements; target words). All eight lists were displayed at the retrieval stage (with the four unselected lists acting as distracter words). This was done to ensure all participants judged the same number of positive and negative words in each condition (self- and other-judged) and in both stages of the task. In both stages, words were displayed sequentially in a random order.

**Table 1:** Word stimuli for the recognition memory task.

**Positive words**							
patient	independent	relaxed	excitable	cautious	restless	calm	likable
talented	energetic	courageous	self-confident	self-critical	daring	popular	persuasive
ambitious	neat	smart	sociable	tidy	polite	optimistic	proud
brave	brilliant	enthusiastic	creative	lively	imaginative	truthful	serious
considerate	kind	happy	intellectual	responsible	cheerful	sensible	easy-going
musical	peaceful	thoughtful	witty	intelligent	trustworthy	friendly	generous
unlucky	scientific	artistic	helpful	honest	interesting	shy	sympathetic
emotional	wealthy	nice	reliable	attractive	open-minded	talkative	clever
quiet	unpredictable	wise	athletic	strong	leader	confident	understanding
**Negative words**							
jealous	greedy	unfriendly	thoughtless	cruel	selfish	impatient	liar
gossipy	annoying	self-centered	careless	nosey	boastful	stupid	untrustworthy
short-tempered	unforgiving	lazy	pessimistic	unreliable	unsympathetic	worrier	unkind
clumsy	unpleasant	insecure	noisy	unhealthy	unimaginative	irresponsible	impolite
unemotional	rebellious	unpopular	unhappy	daydreamer	stubborn	childish	bossy
sarcastic	dishonest	sad	boring	foolish	forgetful	messy	irritating
weak	rude	untidy	moody	plain	lonely	unintelligent	oversensitive

*Note.* For each participant, four lists (four columns in the table) were selected at random to be displayed during the encoding stage (two lists assigned to self-judgement, two lists assigned to other-judgement; target words). The other four lists were used as distractors in the retrieval stage, meaning all eight lists were displayed at the retrieval stage. In both stages, words were displayed sequentially in a random order.

#### 1.2.2 Associative-matching (shapes) task

In the shapes task, participants were instructed that one shape (either a circle or square, counterbalanced between participants) would represent them (self condition) and the other shape would represent their chosen familiar other (other condition).

On each trial, participants were presented with a central fixation cross for 500 ms, followed by the shape-label pair for 100 ms. The shape, either a circle or square, was presented above the fixation cross and the label, either ‘you’ or ‘her’ (selected to match number of letters and stimulus size), was presented underneath the fixation cross. Participants indicated (within 2000 ms) whether the shape and label were a match or not (i.e. if the square represents the self then when square is presented with ‘you’, the correct response is yes) by pressing the left or right arrow key (counterbalanced). Feedback was given through either a smiley or sad face with ‘correct’ or ‘incorrect’ below for 500 ms. Participants were instructed to respond as quickly and as accurately as possible.

The task procedure was adapted from Sui, He, & Humphreys, 2012. The stimuli were optimised for a standard computer monitor size of 22 in., although precise visual angles could not be replicated as participants completed the task on a variety of computer screens (as we tested across three different schools in computer classrooms and in testing cubicles on university campus).

### 1.3 Self-consciousness questionnaire

Participants were asked to complete the Revised Self-consciousness Scale for Children (R-SCS-C; Takishima-Lacasa, Higa-McMillan, Ebesutani, Smith, & Chorpita, 2014). Participants were asked to indicate their agreement with 29 statements on a Likert scale from 1 (‘strongly disagree’) to 5 (‘strongly agree’). These items were analysed using confirmatory factor analysis to extract three factors (replicated from Takishima-Lacasa, Higa-McMillan, Ebesutani, Smith, & Chorpita, 2014): public self-consciousness (representing style and appearance consciousness), private self-consciousness (representing self-reflectiveness and internal state awareness) and social anxiety (see ‘Statistical analysis’ for more detail).

The R-SCS-C showed good internal reliability in our dataset (Cronbach’s alpha:.78, 95% CI [.77.79]). Takishima-Lacasa, Higa-McMillan, Ebesutani, Smith, & Chorpita, (2014) examined the validity of the scale, in a sample of 7–18 year olds, through correlations with the Imaginary Audience Scale (IAS), the Revised Child Anxiety and Depression Scales (RCADS) and the Positive and Negative Affect Schedule for Children (PANAS-C). The R-SCS-C social anxiety and public self-consciousness subscales were significantly correlated with both subscales of the IAS (transient and abiding self), subscales of the RCADS (social phobia, major depression) and both subscales of the PANAS-C (positive and negative affect). Private self-consciousness was significantly correlated with both subscales of the PANAS-C and the RCADS social phobia and major depression subscale (Takishima-Lacasa, Higa-McMillan, Ebesutani, Smith, & Chorpita, 2014).

### 1.4 Matrix reasoning

We included a measure of matrix reasoning as a covariate (in line with previous work, Chierchia, Piera Pi-Sunyer, & Blakemore, 2020) to ensure age-related differences in self-referential processing were not due to differences in non-verbal reasoning ability. To measure non-verbal reasoning ability, the Matrix Reasoning Task (MaRs-IB; Chierchia et al., 2019) was used. In this task, participants are asked to complete an incomplete matrix containing abstract shapes, by choosing the missing shape from four options. The percentage of correct answers was calculated as a measure of non-verbal reasoning ability for each participant and this measure was included as a covariate in all analyses. The advantage of the MaRs-IB task over other non-verbal reasoning tasks is that it is online, open access and short (8 min).

### 1.5 Procedure

**Fig. 2:**
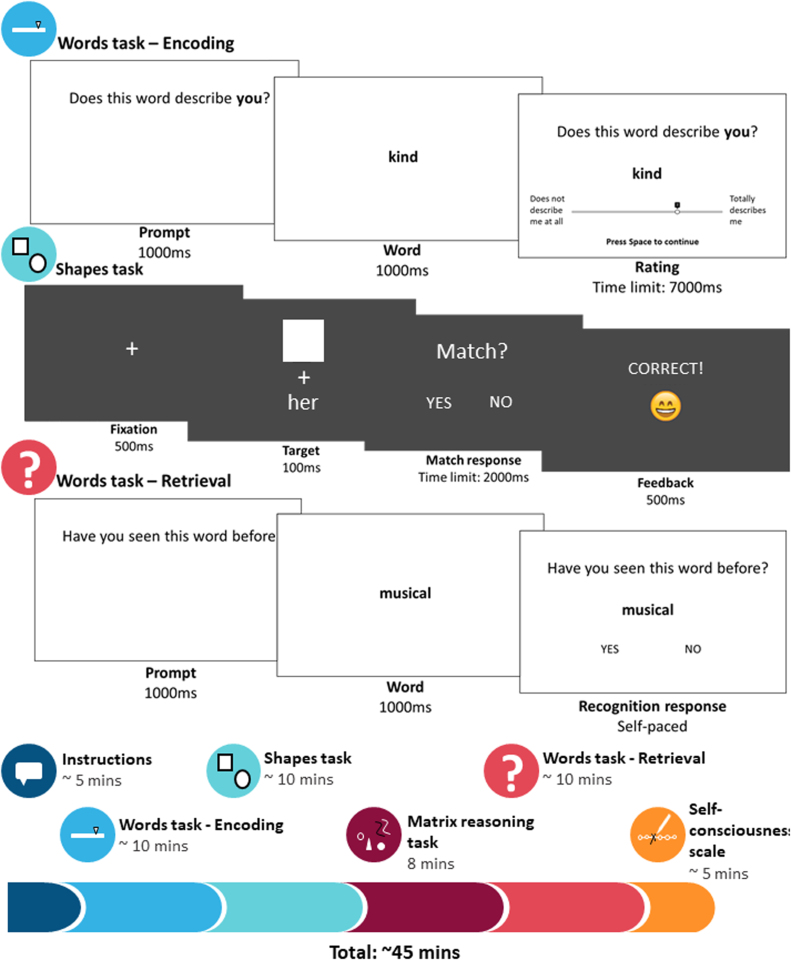
Procedures and timings. *Note*. Words task – Encoding: Participants saw a prompt ‘Does this word describe you/[chosen other]?’ followed by the target word (e.g. kind) and then submitted their rating on a slider from 0 ‘Does not describe me/her at all’ to 10 ‘Totally describes me/her’. Shapes task: Participants saw a fixation cross, followed by the target stimulus (shape-label pair, any combination of circle/square-you/her), and responded to the prompt ‘Match?’ by pressing left or right arrow key for ‘yes’ or ‘no’ (counterbalanced). Participants were given feedback in the form of a smiley or sad face. Words task – Retrieval: Participants saw a prompt ‘Have you seen this word before?’, followed by the target word (e.g. ‘musical’) and responded by pressing the left or right arrow key for ‘yes’ or ‘no’ (counterbalanced). Timeline of procedure (bottom panel): Participants received both verbal and written instructions. They then completed descriptiveness ratings, the shapes task and matrix reasoning task, the surprise recognition memory test and finally the self-consciousness scale.

Participants were tested in groups (testing group size varied from two to 19 and was included as a covariate in all analyses) in a classroom setting (under 18 year olds) or in study cubicles (over 18 year olds). Participants were given verbal instructions at the beginning of the experiment. Participants then entered their age and their chosen ‘familiar other’ person. Participants completed the encoding stage of the recognition memory words task, followed by the associative-matching shapes task and the non-verbal reasoning task (MaRs-IB; which both also acted as distractor tasks for the words task). After this, participants were given the surprise recognition test in the words task (retrieval stage). All tasks were created and hosted on Gorilla.sc. Finally, participants completed the self-consciousness scale (Takishima-Lacasa, Higa-McMillan, Ebesutani, Smith, & Chorpita, 2014) on paper. In total, participants spent approximately 45 mins doing the experiment (See Fig. 2).

### 1.6 Statistical analysis

We used mixed-effects models to analyse our data, which allowed us to use trial-level data (as opposed to aggregate data) and group error terms by participant (Bates et al., 2015).

In the words task, we conducted four separate models for our four dependent variables: descriptiveness ratings (at encoding; see Table 2, Model 1), recognition accuracy at retrieval (Model 2) and reaction times (RTs) at encoding (Model 3) and RTs at retrieval (Model 4). We investigated the relation between these four dependent variables and three independent variables: condition (self- vs other-judged) and word valence (positive vs negative) as within-subject factors, and age, as a between-subjects factor. We also analysed whether descriptiveness ratings predicted recognition accuracy, by including descriptiveness ratings as an additional independent variable (Model 5). Recognition accuracy was a binary variable (coded as 1 for correct and 0 for incorrect) and was assessed for target trials only (but false alarm rate was included as a covariate).

**Table 2:** Model equations.

	**Dependent variable**	**Fixed Effects**	**Random Effects**
	**Words task**		
	***Descriptiveness ratings and recognition accuracy***		
Model 1	Descriptiveness ratings (1–10)	Age^a^ Condition^a^ Valence + MaRs-IB + Testing group size	Condition^a^ Valence
Model 2	Recognition accuracy (1/0)	Age^a^ Condition^a^ Valence + MaRs-IB + FA + Testing group size	Condition^a^ Valence
Model 5	Recognition accuracy (1/0)	Age^a^ Condition^a^ Valence + MaRs-IB + FA + Rating + Testing group size	Condition^a^ Valence
	***RTs***		
Model 3	RTs (log) at encoding	Age^a^ Condition^a^ Valence + MaRs-IB + Testing group size	Condition + Valence
Model 4	RTs (log) at retrieval	Age^a^ Condition^a^ Valence + MaRs-IB + Testing group size	Condition + Valence
	
	***Relationship with self-consciousness scale***		
Models 8–9: Social anxiety, public self-consciousness	Descriptiveness rating (1–10)	Questionnaire factor scores^a^ Condition^a^ Valence + MaRs-IB + Testing group size	Condition + Valence
Model 10: Private self-consciousness	Recognition accuracy (1/0)	Questionnaire factor scores^a^ Condition^a^ Valence + MaRs-IB + Testing group size	Condition + Valence
	**Shapes task**		
	***Accuracy***		
Model 6	Matching accuracy (1/0)	Age^a^ Condition + MaRs-IB + Testing group size	Condition
	***RTs***		
Model 7	RTs (log)	Age^a^ Condition + MaRs-IB + Testing group size	Condition

*Note*. All models used trial-level data. Each model includes the second-degree polynomial of age (i.e. including linear and quadratic functions of age). Condition is a binary variable describing whether stimuli (words and shapes) were self- or other-judged in the words task, and self- or other-associated in the shapes task. Valence is a binary variable describing whether words were positive or negative. MaRs-IB is the proportion correct on the Matrix Reasoning task, included as a covariate to control for age-related differences in reasoning. Testing group size denotes the number of participants in the same testing room. FA is individual level false alarm rate in the words task.

a Indicates interactions for individual terms and all lower-level interactions.

In the shapes task, we conducted two separate models for the two dependent variables: matching accuracy (Model 6) and RTs (Model 7). We investigated the relationship between these dependent variables (matching accuracy and RTs) and two independent variables: condition (self vs other) as a within-subjects factor, and age as a between-subjects factor. Accuracy was a binary variable (coded as 1 for correct, 0 for incorrect) and was assessed for matching judgements only (as in Sui, He, & Humphreys, 2012).

For both tasks, RTs were taken from correct trials only and were log-transformed to better approximate a normal distribution. RTs less than 250 ms were excluded. We included matrix reasoning scores (from MaRs-iB) and testing group size (number of participants in the same testing room) as covariates in all analyses (covariate results can be found in the Supplementary Materials).

We compared various functions of age, namely linear, quadratic, cubic, their combined polynomials (i.e. linear + quadratic, linear + quadratic + cubic), inverse, logarithmic and exponential (Luna, Garver, Urban, Lazar, & Sweeney, 2004), and selected the model with the lowest Akaike Information Criteria (AIC; which penalizes for additional parameters; see Table S1 for AIC model comparison). If this was achieved by a polynomial model (linear + quadratic + cubic), we further compared this to the next lower-level model via nested model comparison (i.e. ANOVA; see Supplementary Materials for statistics; Bates, Mächler, Bolker, & Walker, 2015), progressively removing higher order polynomials if these did not explain significantly more variance. For all outcome variables, this procedure suggested that the second-degree polynomial function of age (i.e. including linear and quadratic functions of age) had the best fit (except for word recognition RTs, where the first-degree polynomial was the best fit).

Trial-level data was modelled using mixed-effects models with the lme4 package in R (Bates et al., 2015, R Core Team, 2014). Ratings and RTs were investigated with linear mixed-effects models, while accuracy was investigated using a generalised linear mixed-effect model and a logit link function (i.e. logistic regression). As fixed effects, we investigated the interaction between, for the words task, the three independent variables (age, condition and target valence) and, for the shapes task, the two independent variables (age and condition) and all lower-level interactions. As random intercepts, we used participant-level IDs. We employed maximal random slopes for the within-subject factors (Barr, 2013). For the RT models in the words task and the self-consciousness factor score models, maximal random slopes led to a singular model fit. This failure to converge is common in models with greater complexity and is generally taken as an indication that the model is over-specified. We thus simplified the random slopes structure by removing the interaction between random slopes for condition and valence (as suggested by Barr, 2013), allowing the model to converge (see Table 2, Models 3-4, 8-10).

We report main effects and interactions of the best-fitting models using omnibus Type III *χ*^2^ Wald tests. These were further probed with planned and post-hoc comparisons using the emmeans package (Lenth, Singmann, Love, Buerkner, & Herve, 2020), which were Bonferroni corrected for multiple comparisons.

#### 1.6.1 Self-consciousness questionnaire

Using confirmatory factor analysis, we obtained participant-level factor scores of a three-factor solution, as reported in Takishima-Lacasa, Higa-McMillan, Ebesutani, Smith, & Chorpita, 2014. These three factors are social anxiety (example: ‘Large groups make me nervous’), public self-consciousness (example: ‘I often check the way I look’) and private self-consciousness (example: ‘I like to understand why I do things’). Confirmatory factor analysis was performed using R lavaan package. Although model test statistics did not reach cut off for good fit (*χ*^2^ (374) = 940.50, *p* < .001; CFI = 0.74; TLI = 0.71; RMSEA = 0.085), a three-factor model was a better fit than a single-factor model or any combination of factors to make a two-factor model according to AIC and Bayesian Information Criteria (BIC; see Table S3). Modification indices were also inspected but did not indicate any modifications that would make a substantial difference to model fit (all values below 10). Factor loadings can be found in the Appendix A, Appendix A.

We explored associations between factor scores and our task measures by including factor scores as a DV in mixed-models (replacing age; see Table 2, Models 8–10).

## 2 Results

### 2.1 Familiarity and likeability of chosen other

Participants tended to choose people with whom they were familiar (mean (SD) familiarity rating = 6.31 (2.26); max score 10) and who they liked (mean (SD) likeability rating = 8.21 (1.77); max score 10). Likeability and familiarity ratings were not correlated with participant age (likeability: *r* = −0.101, *p* = .143; familiarity: *r* = −0.079, *p* = .253). Participants chose fictional characters (107 participants) or public figures e.g. singers, actors, youtubers etc. (79 participants; 24 unidentifiable as only first name given).

### 2.2 Recognition memory (words) task

#### 2.2.1 Encoding - descriptiveness ratings

Participants rated positive words overall as more descriptive than negative words (main effect of valence: *χ*^2^ (1) = 792.84, *p* < .001, contrast_Pos-Neg_ = 2.90, SE = 0.1, *p* < .001). There was an interaction between age and valence (*χ*^2^ (1) = 5.86, *p* = .015), in that the tendency to rate positive words as descriptive showed an age-related linear increase (slope_Pos_ = 22.23, *SE* = 7.0, *p* = .001) whereas the tendency to rate negative words as descriptive stayed relatively stable with age (slope_Neg_ = −6.69, *SE* = 9.0, *p* = .458; contrast_Pos–Neg_ = 28.90, *SE* = 11.9, *p* = .016).

**Fig. 3:**
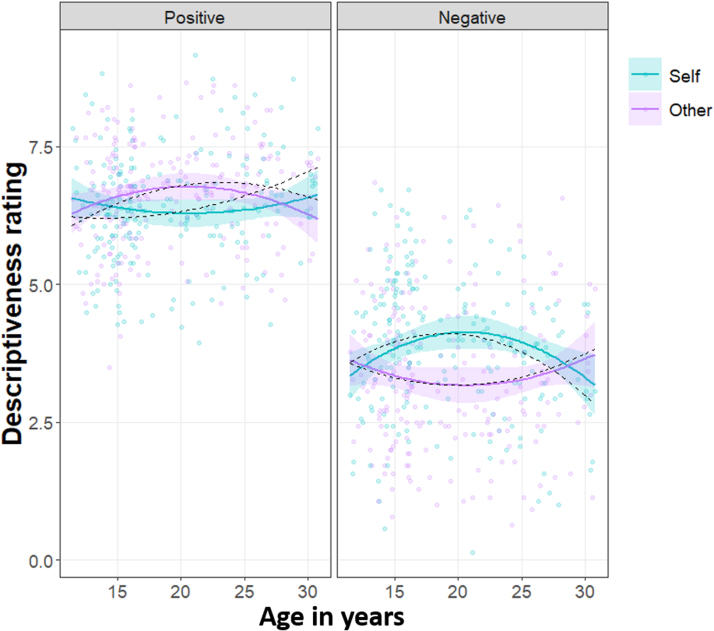
Self- and other-judged descriptiveness ratings for positive versus negative words plotted against age in years. *Note*. Participants rated words on a scale from 0 to 10. Solid coloured lines represent significant quadratic component of age (ribbons: 95% confidence interval), dotted black lines represent estimated fixed effects for the polynomial effect of age; data points are participants’ mean descriptiveness ratings.

There was a three-way interaction between the quadratic component of age, valence and condition (*χ*^2^ (1) = 11.66, *p* = .001; Fig. 3). Inspection of model estimates revealed that there was an age-related increase in negative self-judgements between 11 and 19 years, which peaked at around 19 years, and was followed by an age-related decrease across the 20 s. This was shown in the negative quadratic association (inverted U-shaped curve) between age and ratings of negative self-judged words (Prediction 2; slope_Self (Neg)_ = −25.99, *SE* = 9.8, *p* = .008; Fig. 3 right panel). This was not the case for ratings of negative other-judged words (slope_Other (Neg)_ = 15.40, *SE* = 10.7, *p* = .149; contrast_Self–Other (Neg)_ = −41.39, *SE* = 12.1, *p*_Bonf_ = .001; Fig. 3 right panel). There was also a negative quadratic association (inverted U-shaped curve) between age and the tendency to rate positive words as more descriptive of the other (peaking at age 23.2) but not the self (slope_Other (Pos)_ = −15.91, *SE* = 7.7, *p* = .038; slope _Self (Pos)_ = 9.14, *SE* = 8.0, *p* = .254; contrast_Self – Other (Pos)_ = 25.05, *SE* = 10.3, *p*_Bonf_ = .030; Fig. 3 left panel).

#### 2.2.2 Encoding – reaction times

Participants were faster at rating descriptiveness of words for the familiar other than for themselves (main effect of condition: *χ*^2^(1) = 9.38, *p* = .002; contrast_Self-Other_ = 0.03, SE = 0.01, *p* = .002) and positive compared with negative words (main effect of valence (*χ*^2^(1) = 105.07, *p* < .001; contrast_Pos–Neg_ = −0.08, SE = 0.01, *p* < .001). There was a significant effect of both components of age (main effect of age: linear *χ*^2^(1) = 5.31, *p* = .021, estimate = −9.23, *SE* = 4.01, *p* = .022; main effect of age: quadratic *χ*^2^(1) = 16.54, *p* < .001, estimate = 11.62, *SE* = 2.86, *p* < .001), showing an age-related decrease in descriptiveness ratings RTs that plateaued around age 22.

#### 2.2.3 Retrieval - recognition accuracy

Memory performance overall remained stable across age (main effect of age: linear *χ*^2^ (1) = 0.77, *p* = .379; quadratic *χ*^2^ (1) = 1.92, *p* = .166).

**Fig. 4:**
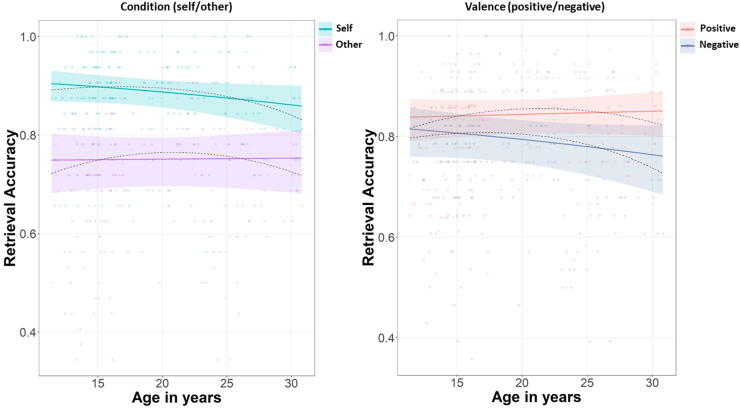
Recognition accuracy plotted against age in years. *Note*. Recognition accuracy plotted against age in years. Left panel: Recognition accuracy for self- and other-judged words against age (collapsed across positive and negative words). Right panel: Recognition accuracy for positive and negative words against age (collapsed across self- and other-judged). For both figures, solid lines represent the significant linear component of age (ribbons: 95% confidence interval); dotted lines represent the estimated fixed effects for the polynomial effect of age; data points are participants’ proportion recognition accuracy.

There was a significant self-reference effect (SRE) overall, as all participants correctly recognised more self-judged than other-judged words (main effect of condition, self vs other: *χ*^2^ (1) = 328.86, *p* < .001; contrast_Self – Other_ = 1.02, *SE* = 0.1, *p* < .001) and the size of the SRE reduced linearly with age (Prediction 1; interaction linear age and condition: *χ*^2^ (1) = 4.29, *p* = .038; Fig. 4 left panel), such that memory for self-judged words decreased linearly and memory for other-judged words increased linearly across our age range (11–30 years; slope_Self_ = −22.12, *SE* = 14.3, *p* = .122; slope_Other_ =0.27, *SE* = 12.8, *p* = .983; contrast_Self-Other_ = −22.4, *SE* = 10.8, *p* = .038).

There was a significant positivity memory bias overall, as all participants correctly recognised more positive than negative words (main effect of valence: *χ*^2^ (1) = 43.19, *p* < .001; contrast_Pos – Neg_ = 0.35, *SE* = 0.05, *p* < .001). This positivity bias in memory increased linearly with age (interaction age and valence: *χ*^2^ (1) = 7.32, *p* = .007; Fig. 4 right panel), such that memory for negative words decreased and memory for positive words increased across our age range (11–30 years; slope_Pos_ = 3.08, *SE* = 13.0, *p* = .813; slope_Neg_ = −24.93, *SE* = 13.9, *p* = .073; contrast_Pos-Neg_ = 28.0, *SE* = 10.4, *p* = .007).

**Fig. 5:**
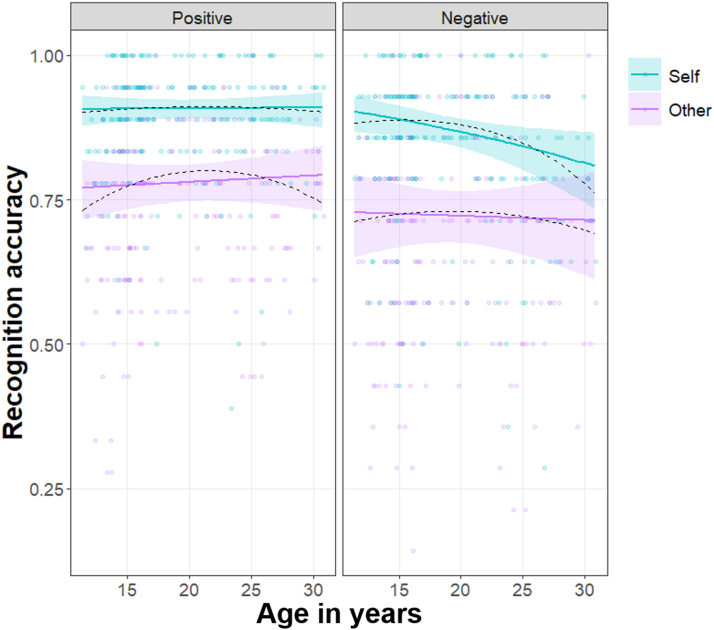
Recognition accuracy for self- and other-judged positive versus negative words plotted against age in years. *Note*. Solid lines represent significant linear component of age (ribbons: 95% confidence interval); dotted lines represent estimated fixed effects of polynomial age; data points are participants’ mean recognition accuracy.

Finally, recognition of self-judged negative words specifically, appeared to decrease linearly across the age range included (Fig. 5). Although the three-way interaction between the linear component of age, valence and condition did not reach significance (Prediction 3; *χ*^2^ (1) = 3.63, *p* = .057), we conducted post-hoc exploratory comparisons to compare slopes for negative versus positive self- and other-judged words. For negative words, memory for self-judged words decreased linearly with age but memory for other-judged words stayed relatively stable (slope_Self (Neg)_ = −44.67, *SE* = 16.3, *p* = .006; slope_Other (Neg)_ = −5.20, *SE* = 14.6, *p* = .723; contrast_Self–Other (Neg)_ = −39.47, *SE* = 13.7, *p*_Bonf_ = .008; Fig. 5 right panel). In contrast, there was no age-related change in memory for either self-judged or other-judged positive words (slope_Self (Pos)_ = 0.42, *SE* = 16.3, *p* = .979; slope_Other (Pos)_ = 5.73, *SE* = 13.3, *p* = .667; contrast_Self–Other (Pos)_ = −5.32, *SE* = 14.4, *p*_Bonf_ = 1; Fig. 5 left panel). However, since the three-way interaction was not significant (*p* = .057) these results should be interpreted with caution.

When including descriptiveness ratings as an additional predictor in the mixed-effects model, descriptiveness ratings significantly predicted recognition accuracy (*χ*^2^ (1) = 138.82, *p* = .001), suggesting that words rated as more descriptive by participants were more likely to be remembered across all ages. All effects reported above remained significant in this model, except for the main effect of valence (main effects: condition *χ*^2^ (1) = 317.26, *p* < .001; valence *χ*^2^ (1) = 1.09, *p* = .297; interactions: age-by-condition *χ*^2^ (1) = 3.87, *p* = .049; age-by-valence *χ*^2^ (1) = 5.14, *p* = .023; age-by-condition-by-valence *χ*^2^ (1) = 2.28, *p* = .131).

#### 2.2.4 Retrieval – reaction times

Participants were faster to correctly recognise self-judged words compared with other-judged words (main effect of condition: *χ*^2^ (1) = 119.44, *p* < .001; contrast_Self–Other_ = −0.19, SE = 0.02, *p* < .001) and positive compared with negative words (main effect of valence: *χ*^2^ (1) = 31.62, *p* < .001; contrast _Pos – Neg_ = −0.09, SE = 0.02, *p* < .001). There were no other significant main effects or interactions.

### 2.3 Associative-matching (shapes) task

Overall, participants showed the SRE in both accuracy and RTs in the shapes task. Participants were more accurate (main effect of condition: *χ*^2^ (1) = 5.58, *p* = .018; contrast_Self–Other_ = 0.24, *SE* = 0.1, *p* = .018) and faster to make matching decisions (main effect of condition: *χ*^2^ (1) = 14.05, *p* < .001, contrast_Self–Other_ = −0.05, *SE* = 0.01, *p* < .001) for self-associated compared with other-associated shapes.

There was no change in the size of the SRE with age in either accuracy (interaction condition and age: linear *χ*^2^ (1) = 0.44, *p* = .507; quadratic *χ*^2^ (1) = 0.81, *p* = .368) or RTs (interaction condition and age: linear *χ*^2^ (1) = 2.12, *p* = .145; quadratic *χ*^2^ (1) = 0.07, *p* = .797).

### 2.4 Relationship between words and shapes SRE

The size of the SRE (mean accuracy for self – mean accuracy for other) for the words and the shapes task were not associated with one another (*r* = −0.01, *p* = .865).

### 2.5 Self-consciousness, social anxiety and self-referential processing of evaluative stimuli

#### 2.5.1 Descriptiveness ratings

Heightened social anxiety was related to increased negative and decreased positive judgements about the self but not the other (Prediction 4; interaction social anxiety, condition and valence: *χ*^2^ (1) = 38.96, *p* < .001; post-hoc contrasts: slope_Self (Pos)_ = −0.31, *SE* = 0.1, *p* < .001, slope_Other (Pos)_ = −0.06, *SE* = 0.1, *p* = .364, contrast_Self–Other (Pos)_ = −0.25, SE = 0.1, *p*_Bonf_ < .001; slope_Self (Neg)_ = 0.42, *SE* = 0.1, *p* < .001, slope_Other (Neg)_ = 0.06, *SE* = 0.1, *p* = .432; contrast_Self–Other (Neg)_ = 0.35, *SE* = 0.1, *p*_Bonf_ < .001). Social anxiety showed a quadratic negative relationship with age (β = −0.15, *SE* =0.1, *p* = .034).

Public self-consciousness (style and appearance consciousness) was related specifically to increased negative self-judgements (Prediction 4; interaction public self-consciousness, condition and valence: *χ*^2^ (1) = 4.51, *p* = .034; post-hoc contrasts: slope_Self (Neg)_ = 0.21, *SE* = 0.1, *p* = .015, slope_Other (Neg)_ = 0.01, *SE* = 0.1, *p* = .859, contrast_Self–Other (Neg)_ = 0.19, *SE* = 0.1, *p*_Bonf_ = .018). Public self-consciousness showed a quadratic negative relationship with age (β = −0.21, *SE* =0.1, *p* = .002).

#### 2.5.2 Self-reference effect

We assessed whether private self-consciousness (self-reflectiveness and internal state awareness) was associated with an increased self-reference effect in the recognition memory task. There was no interaction between private self-consciousness and condition (Prediction 5; χ^2^ (1) = 0.03, p = .855).

## 3 Discussion

In the current study, females aged 11–30 years, completed two self-referential tasks: a recognition memory task employing evaluative words and an associative-matching task employing neutral shapes. Participants also self-reported their levels of self-consciousness and social anxiety. When stimuli were evaluative (words task), the size of the self-reference effect (SRE), namely the tendency to recognise more self-judged words than other-judged words, showed an age-related decrease. When stimuli were neutral (shapes task), there was no age-related change in the size of the SRE, the tendency to make more accurate judgements of self-associated compared to other-associated shapes. In addition, the tendency to judge negative words as descriptive of the self increased across adolescence, peaking around 19 years, before reducing again in early adulthood. This tendency to rate negative words as descriptive of the self was associated with heightened social anxiety and public self-consciousness. In recognition memory, the magnitude of the positivity bias, the tendency to recognise more positive than negative words, increased across the age range included (11–30 years).

### 3.1 Self-reference effect across age

Our first prediction, that the SRE would reduce with age, was partially supported by the data. The SRE reduced linearly with age when stimuli were evaluative (in the words task) but not when stimuli were neutral (in the shapes task). The youngest participants in this study (11-year-olds) had recently transitioned to secondary school. This period of transition is associated with major changes to peer networks (Brown, 2013) as well as pubertal changes (Schaffhuser, Allemand, & Schwarz, 2017, Simmons et al., 1979) and development in the ability to reflect on the self and others Dumontheil, Apperly, & Blakemore, 2010, Moses-Payne et al., 2021, Pfeifer et al., 2009, Symeonidou et al., 2016. During this period of transition, adolescents strive to become independent from their parents and discover their own identity (Koepke & Denissen, 2012). This may elicit uncertainty in or interrogation of the self-concept (Cole et al., 2001). We suggest that this uncertainty might motivate increased self-referential processing, meaning that self-relevant information is more salient and more likely to be remembered. Adolescents may gradually become more certain about their self-concept and therefore show reduced self-referential processing. This might help to explain why we observed an SRE in the words but not the shapes task. The self-relevant words in the recognition memory task may be particularly salient to younger participants as these descriptors can be used to inform their self-concept. In contrast, neutral stimuli arbitrarily associated with the self, as in the shapes task, are not informative and thus might not be particularly salient to the younger participants.

Interestingly, we found that memory performance in general (ignoring condition or valence) remained stable across our age range. It might be assumed that memory performance would improve across adolescence and into early adulthood, although, this is not consistently reported in the literature (Ghetti & Angelini, 2008, Pauls et al., 2013) and social and self-related cognitive processes have shown nonlinear development with age (Diamond, Carey, & Back, 1983, Moses-Payne et al., 2021, Somerville et al., 2013). In the case of self-referential memory, the affective and motivational nature of the task might mean that the younger participants in our study were able to reach a better memory performance than under other encoding conditions.

### 3.2 Negative self-judgements across age

The use of evaluative stimuli in the recognition memory task allowed us to assess how valence influences self-referential processing in adolescence and into adulthood in female participants. Consistent with previous research and our second prediction, we showed that negative self-judgements increased across adolescence (peaking at around age 19) and decreased into adulthood. Several explanations for this increase in negative self-judgements across adolescence have been proposed. First, adolescents might transition from relying on parental judgements in childhood, to leaning more on potentially less favourable peer judgements to inform their self-judgements (Brown, 2013, van der Aar et al., 2018). Consistent with this, adolescents tend to endorse the most positive attributes about themselves when taking the perspective of their mother rather than of their friends or their own perspective (Pfeifer et al., 2009). Further, adolescents’ self-judgements are harsher when they are asked to compare themselves with a peer, especially in 15–17 year olds (van der Aar et al., 2018).

In the current study, we found that negative self-judgements were associated with increased social anxiety and public self-consciousness. Again, this highlights the importance of the looking-glass self, that is, our self-identity when taking the perspective of others (Cooley, 1902) in self-concept development. Further, we found an age-related increase in positive judgements about the chosen other across adolescence, which peaked at around age 23 and was followed by an age-related decrease in the late twenties. If individuals have an inflated positive view of others, this might additionally explain why comparisons with others negatively impacts on self-judgements. In future, it will be important to further categorise descriptive words into domains. Previous work has shown that the trajectory of positive and negative self-judgements across adolescence may depend on domain, for example, whether participants are asked to judge their academic, social or physical traits (Preckel, Niepel, Schneider, & Brunner, 2013, van der Aar et al., 2018).

The results of the current study showed an age-related inverted U-shaped curve in negative self-judgements, but did not show the opposite age-related change (i.e. U-shaped curve) in positive self-judgements. This suggests that the positive and negative domain in self-judgements might be independent. This specific age-related change in negative, but not positive, self-judgements has been shown previously (McArthur et al., 2019) and echoes work suggesting that individuals’ experience of positive and negative affect may be independent of each other (Diener & Emmons, 1984, Watson, Clark, & Tellegen, 1988).

### 3.3 Positivity memory bias across age

Here, we extended previous work by assessing recognition memory for self- and other-judged positive and negative words. This led to our third prediction, that memory for self-judged negative words would increase across adolescence and then decrease in early adulthood. We found that participants of all ages showed a positivity bias in recognition memory, remembering more positive words compared with negative words. The magnitude of this positivity memory bias increased linearly with age, in that younger participants remembered more negative and fewer positive words than did older participants.

This age-related increase in positivity memory bias suggests that positive person-relevant trait words become more salient and negative person-relevant trait words become less salient across adolescence and early adulthood in female participants. Previous work on the positivity memory bias in adolescence has been mixed, with some authors suggesting a negativity bias (Bone et al., 2021, Quas et al., 2016) and others showing different valence biases according to whether free recall or recognition was assessed (Neshat-Doost, Taghavi, Moradi, Yule, & Dalgleish, 1998). Here, we showed that, when words were encoded through self- and other-judgements, there was an age-related increase in the positivity memory bias. This positivity bias may reflect a motivation to uphold a positive view of the self and others (i.e. self-serving bias, Miller & Ross, 1975; or an optimism bias, Sharot, 2011), which may develop across adolescence according to our results.

A reduced positivity bias in (self-referential) memory has previously been shown to be associated with depressive symptoms during adolescence Bone et al., 2021, Connolly, Abramson, & Alloy, 2016, Hammen & Zupan, 1984. Adolescents, relative to other age groups, are at heightened risk of developing mental health conditions such as depression and the reason for this heightened risk is still not well understood (Kessler et al., 2007). Further, low self-esteem has been shown to prospectively predict onset of depression in adolescence (Orth, Robins, & Roberts, 2008, Rieger, Göllner, Trautwein, & Roberts, 2016) and self-hatred has been shown to be a central symptom in adolescent depression (Mullarkey, Marchetti, & Beevers, 2019). Developing a positive self-concept during adolescence may mediate mental health consequences (Neff & McGehee, 2010). Therefore, future work should aim to assess how emerging self-referential processes and memory biases are associated with the onset of depressive symptoms during this critical period of development.

### 3.4 Limitations and future directions

We recruited a female only sample and our results might not generalise to males. It is unclear whether there are consistent gender differences in self-referential processing. Previous studies on gender differences in the self-reference effect (Bentley, Greenaway, & Haslam, 2017) and on self-judgements (Jacobs, Lanza, Osgood, Eccles, & Wigfield, 2002, McArthur et al., 2019, van Buuren et al., 2020) have been mixed. In other studies, gender differences were not investigated or a single-gender sample was recruited (Ray et al., 2009) as in the current study. Therefore, further work is needed to investigate whether the results found here replicate in males.

Another limitation of our study was that verbal word span was not measured. Verbal word span may affect memory performance on the recognition memory task overall. However, words were randomly assigned to target (shown at encoding, to-be-remembered) and distractors (shown only at retrieval), and randomly split across the self and other-judged conditions. Therefore, we would not expect verbal word span to have a significant impact on memory performance *between* our factors of interest.

In previous research, participants were typically assigned a famous figure or fictional character to give descriptiveness ratings about Dégeilh et al., 2015, Pfeifer et al., 2013, Pfeifer, Lieberman, & Dapretto, 2007 or participants were asked to answer about a relative or a peer (Ray et al., 2009, van der Aar et al., 2018). Here, we asked participants to choose a famous person or fictional character themselves, in order to ensure that they were familiar with them and to increase engagement with the task (we assumed participants would be more interested to answer about someone of their choosing). We asked participants to rate the familiarity and likeability of the person they chose and showed that participants reported being familiar with and liking the person they chose. We were also able to report the profession of the person chosen or whether they were a fictional character. However, we did not ask any further details about the participants’ views of their chosen person and future work should assess how the effects reported here are mediated by factors such as how similar the participant feels to or whether they admire their chosen person.

### 3.5 Conclusions

Through adolescence, young people seek to construct their self-concept. We found that, when stimuli were evaluative, the self-reference effect decreased across the age range included in the study (11–30 years). When stimuli were neutral, the self-reference effect remained stable across age. We found that valence was an important factor in females’ self-referential processing. The tendency to rate negative words as descriptive of the self showed an age-related increase across adolescence, peaking around 19 years and decreasing in early adulthood. Finally, our results demonstrated an age-related increase in the magnitude of a positivity bias in memory. These findings suggest that adolescents may have cognitive biases that shift their attention towards information that can help them construct a sense of self but might be particularly focussed on negative aspects of themselves and others. Further research is needed to understand why self-judgements become increasingly negative in adolescence and how memory biases towards negative stimuli impact on self-concept development across the lifespan.

## CRediT authorship contribution statement

MEMP and SJB designed the study. MEMP wrote the manuscript with help from SJB and GC. MEMP collected and analysed data. GC wrote the original analysis scripts. This work has been seen and reviewed by all authors.

Data will be made available in a public repository on acceptance.

The authors declare no competing financial interests.

The study was approved by the UCL Research Ethics Committee (Project ID: 3453/001).
