# Supplementary material for: Age-related changes in the impact of valence on self-referential processing in female adolescents and young adults
Source: Cogn Dev. 2022 Jan-Mar;61:None. doi: 10.1016/j.cogdev.2021.101128 (PMC8791274; doi:10.1016/j.cogdev.2021.101128)
Supplement: Supplementary file 1 — Supplementary material [file mmc1.docx]

**Supplementary Materials**

**Model comparison – forms of age**

**Table S1**

*Model comparison of different forms of age for each task outcome.*

|  | **Akaike Information Criterion (AIC)** | | | | | |
| --- | --- | --- | --- | --- | --- | --- |
|  | **Words task** | | | | **Shapes task** | |
| **Form of age** | *Descriptiveness ratings* | *Recognition accuracy* | *Descriptiveness ratings (RT)* | *Recognition (RT)* | *Matching Accuracy* | *Matching RT* |
| Age | 63737.35 | 13138.47 | 16322.60 | 21409.57 | 8648.97 | 9090.90 |
| e^Age^ | 64009.59 | 13145.95 | 16599.83 | 21679.06 | 8664.68 | 9230.04 |
| log(Age) | 63766.48 | 13138.33 | 16347.15 | 21436.46 | 8646.34 | 9100.54 |
| Age^-1^ | 63744.68 | 13138.02 | 16320.89 | 21413.59 | 8643.96 | 9084.27 |
| Age^2^ | 63817.08 | 13138.50 | 16406.45 | 21488.84 | 8652.15 | 9134.42 |
| Age^3^ | 63843.93 | 13138.51 | 16436.55 | 21516.23 | 8655.11 | 9150.51 |
| Age+Age^2^ | 63713.19 | **13122.22** | **16307.17** | 21405.31 | **8641.49** | 9058.68 |
| Age+Age^2^ +Age^3^ | **63694.73** | 13138.02 | 16308.03 | **21405.26** | 8643.81 | **9054.15** |

*Note.* Lower AIC values indicate a better fitting model (bold values indicate the lowest AIC for different model outcomes).

We compared various functions of age (see column one in Table S1) and selected the model with the lowest Akaike Information Criteria (AIC). If this was achieved by a polynomial model (e.g. Age + Age^2^ + Age^3^), we further compared this to the next lower-level model via nested model comparison (i.e. ANOVA; Bates et al., 2015).

For the words task descriptiveness ratings outcome, the third-degree polynomial (Age + Age^2^ + Age^3^) was not a significantly better fit than the second-degree polynomial (Age + Age^2^; *χ*^2^(4) = 7.96, p = .093) but the second-degree polynomial was a significantly better fit than the first-degree polynomial (*χ*^2^(4) = 13.66, p = .008). Therefore, we selected the second-degree polynomial model as our best fitting model.

For the words task recognition accuracy outcome, the third-degree polynomial was not a significantly better fit than the second-degree polynomial (*χ*^2^(4) = 7.96, p = .093) but the second-degree polynomial was a significantly better fit than the first-degree polynomial (*χ*^2^(4) = 13.66, p = .008). Therefore, we selected the second-degree polynomial model as our best fitting model.

For the descriptiveness ratings RT outcome, the second-degree polynomial was a significantly better fit than the first-degree polynomial (*χ*^2^(4) = 18.76, p < .001). Therefore, we selected the second-degree polynomial model as our best fitting model.

For the recognition accuracy RT outcome, the third-degree polynomial was not a significantly better fit than the second-degree polynomial (*χ*^2^(4) = 1.10, p = .893) and the second-degree polynomial was not a significantly better fit than the first-degree polynomial (*χ*^2^(4) = 5.34, p = .254). Therefore, we selected the first-degree polynomial model as our best fitting model.

For the shapes task matching accuracy outcome, the second-degree polynomial was a significantly better fit than the first-degree polynomial (*χ*^2^(4) = 11.48, p = .003). Therefore, we selected the second-degree polynomial model as our best fitting model.

For the matching RT outcome, the third-degree polynomial was not a significantly better fit than the second-degree polynomial (*χ*^2^(4) = 4.72, p = .094) but the second-degree polynomial was a significantly better fit than the first-degree polynomial (*χ*^2^(4) = 32.65, p < .001). Therefore, we selected the second-degree polynomial model as our best fitting model.

**Effect of Covariates: Matrix Reasoning and Testing Group Size**

***Matrix Reasoning***

There was no significant effect of matrix reasoning on descriptiveness ratings. Matrix reasoning ability was associated with better recognition memory (*χ*^2^ (1) = 9.78, *p* = .002, *estimate* = .14, *SE* = .06, *p* = .002). The effect of matrix reasoning was not significant in any other models in the recognition memory task.

In the shapes task, better matrix reasoning ability was associated with better matching accuracy (*χ*^2^ (1) = 5.58, *p* = .018, *estimate* = .27, *SE* = .05, *p* < .001) and faster reaction times (*χ*^2^ (1) = 18.54, *p* < .001, *estimate* = -.05, *SE* = .01, *p* < .001).

The effect of matrix reasoning was not significant in any other models.

***Testing Group Size***

There was a significant effect of testing group size in the descriptiveness ratings model (*χ*^2^ (1) = 4.81, *p* = .028), those who were tested in larger testing group sizes tended to rate words as more descriptive overall than those tested in smaller testing group sizes (*estimate* = .10, *SE* = .05, *p* = .029). The effect of testing group size was not significant in any other models.

**Table S2**

*Word stimuli with likeability ratings, social desirability ratings, positive versus negative categorisation and age of acquisition.*

|  | **Likeability (Anderson, 1968)** | **Social desirability (Kirby & Gardner, 1972)** | **Positive (1) Negative (0)** | **Age of Acquisition (Brysbaert & Biemillier, 2017)** |
| --- | --- | --- | --- | --- |
| patient | 478 |  | 1 | 4 |
| talented | 478 |  | 1 |  |
| ambitious | 484 |  | 1 | 6 |
| brave |  | 6.83 | 1 | 2 |
| considerate | 527 |  | 1 | 6 |
| musical |  | 5.97 | 1 | 2 |
| unlucky | 280 |  | 1 | 2 |
| emotional | 283 |  | 1 | 8 |
| quiet | 311 |  | 1 | 2 |
| jealous | 104 |  | 0 | 4 |
| gossipy | 119 |  | 0 |  |
| short-tempered | 159 |  | 0 |  |
| clumsy | 199 |  | 0 | 6 |
| unemotional | 209 |  | 0 |  |
| sarcastic | 210 |  | 0 | 8 |
| weak |  | 2.87 | 0 | 4 |
| independent | 455 |  | 1 | 6 |
| energetic | 457 |  | 1 | 6 |
| neat | 466 |  | 1 | 2 |
| brilliant | 490 |  | 1 | 8 |
| kind | 520 |  | 1 | 2 |
| peaceful |  | 7.43 | 1 | 2 |
| scientific |  | 5.86 | 1 | 4 |
| wealthy |  | 5.68 | 1 | 4 |
| unpredictable | 290 |  | 1 | 8 |
| greedy | 72 |  | 0 | 2 |
| annoying | 84 |  | 0 |  |
| unforgiving | 98 |  | 0 |  |
| unpleasant | 104 |  | 0 | 4 |
| rebellious | 258 |  | 0 | 8 |
| dishonest | 41 |  | 0 | 4 |
| rude | 76 |  | 0 | 4 |
| relaxed | 439 |  | 1 |  |
| courageous | 471 |  | 1 | 4 |
| smart | 488 |  | 1 | 2 |
| enthusiastic | 489 |  | 1 | 6 |
| happy | 514 |  | 1 | 2 |
| thoughtful | 529 |  | 1 | 2 |
| artistic |  | 6.53 | 1 | 4 |
| nice |  | 6.52 | 1 | 2 |
| wise |  | 7.53 | 1 | 2 |
| unfriendly | 92 |  | 0 | 4 |
| self-centered | 96 |  | 0 |  |
| lazy | 126 |  | 0 | 2 |
| insecure | 198 |  | 0 | 4 |
| unpopular | 222 |  | 0 | 4 |
| sad | 209 |  | 0 | 2 |
| untidy | 175 |  | 0 | 6 |
| excitable | 317 |  | 1 | 4 |
| self-confident | 421 |  | 1 |  |
| sociable | 429 |  | 1 | 8 |
| creative | 462 |  | 1 | 8 |
| intellectual | 476 |  | 1 | 10 |
| witty | 480 |  | 1 | 6 |
| helpful | 492 |  | 1 | 2 |
| reliable | 527 |  | 1 | 6 |
| athletic |  | 6 | 1 | 4 |
| thoughtless | 77 |  | 0 | 2 |
| careless | 140 |  | 0 | 2 |
| pessimistic | 164 |  | 0 | 8 |
| noisy | 173 |  | 0 | 2 |
| unhappy | 203 |  | 0 | 2 |
| boring | 97 |  | 0 |  |
| moody | 182 |  | 0 | 8 |
| cautious | 334 |  | 1 | 6 |
| self-critical | 389 |  | 1 |  |
| tidy | 427 |  | 1 | 6 |
| lively | 466 |  | 1 | 2 |
| responsible | 505 |  | 1 | 4 |
| intelligent | 537 |  | 1 | 6 |
| honest | 555 |  | 1 | 4 |
| attractive |  | 7 | 1 | 4 |
| strong |  | 6.6 | 1 | 2 |
| cruel | 40 |  | 0 | 2 |
| nosey | 102 |  | 0 |  |
| unreliable | 104 |  | 0 | 4 |
| unhealthy | 197 |  | 0 | 2 |
| daydreamer | 260 |  | 0 |  |
| foolish |  | 2.63 | 0 | 2 |
| plain |  | 4.1 | 0 | 4 |
| restless | 274 |  | 1 | 6 |
| daring | 360 |  | 1 | 4 |
| polite | 489 |  | 1 | 2 |
| imaginative | 492 |  | 1 | 8 |
| cheerful | 504 |  | 1 | 2 |
| trustworthy | 539 |  | 1 | 6 |
| interesting |  | 7.34 | 1 | 2 |
| open-minded |  | 7.93 | 1 |  |
| leader |  | 6.67 | 1 |  |
| selfish | 82 |  | 0 | 4 |
| boastful | 122 |  | 0 | 6 |
| unsympathetic | 153 |  | 0 |  |
| unimaginative | 195 |  | 0 |  |
| stubborn | 196 |  | 0 | 4 |
| forgetful | 224 |  | 0 | 2 |
| lonely | 256 |  | 0 | 2 |
| calm | 406 |  | 1 | 6 |
| popular | 426 |  | 1 | 4 |
| optimistic | 443 |  | 1 | 10 |
| truthful | 454 |  | 1 | 2 |
| sensible | 464 |  | 1 | 4 |
| friendly | 519 |  | 1 | 2 |
| shy | 291 |  | 1 | 2 |
| talkative | 352 |  | 1 | 2 |
| confident | 401 |  | 1 | 8 |
| impatient |  | 3.2 | 0 |  |
| stupid |  | 2.23 | 0 | 4 |
| worrier | 205 |  | 0 | 2 |
| irresponsible | 106 |  | 0 | 6 |
| childish | 109 |  | 0 | 4 |
| messy | 147 |  | 0 | 2 |
| unintelligent | 168 |  | 0 |  |
| likable | 497 |  | 1 | 8 |
| persuasive | 374 |  | 1 | 8 |
| proud | 358 |  | 1 | 2 |
| serious | 379 |  | 1 | 6 |
| easygoing | 412 |  | 1 | 8 |
| generous | 459 |  | 1 | 6 |
| sympathetic | 459 |  | 1 | 6 |
| clever | 496 |  | 1 | 4 |
| understanding | 549 |  | 1 | 2 |
| liar | 26 |  | 0 | 2 |
| untrustworthy | 43 |  | 0 |  |
| unkind | 66 |  | 0 | 2 |
| impolite | 103 |  | 0 | 4 |
| bossy | 112 |  | 0 | 4 |
| irritating | 118 |  | 0 |  |
| oversensitive | 179 |  | 0 |  |

*Note.* A median split on Anderson (1968) likeability ratings was used to categorise words into positive or negative. Where likeability ratings were not available, social desirability ratings from Kirby & Gardner (1972) were used in the same way.

**Table S3**

*Model comparison in self-consciousness scale confirmatory factor analysis.*

| Model | AIC | BIC | Sample-size adjusted BIC |
| --- | --- | --- | --- |
| Three-factor (Takishima-Lacasa et al., 2014) | **17067.00** | **17271.17** | **17077.89** |
| One-factor | 17942.83 | 18136.96 | 17953.18 |
| Two-factor (public and social anxiety factors combined) | 17451.11 | 17648.59 | 17461.64 |
| Two-factor (public and private combined) | 17566.72 | 17764.20 | 17577.25 |
| Two-factor (private and social anxiety combined) | 17461.83 | 17659.31 | 17472.37 |

*Note.* A three-factor model structure from Takishima-Lacasa et al., (2014), in bold, was the winning model with the lowest AIC, BIC and sample-size adjusted BIC.

**Table S4**

*Factor loadings for each self-consciousness scale questionnaire item.*

|  | Public | Private | Social Anxiety |
| --- | --- | --- | --- |
| I worry about the way I look | 0.571 |  |  |
| I notice my inner feelings a lot |  | 0.561 |  |
| Large groups make me nervous |  |  | 0.622 |
| I often check the way I look | 0.715 |  |  |
| I know how my body reacts to certain feelings |  | 0.402 |  |
| I know how my feelings affect how I act |  | 0.399 |  |
| I feel scared when I have to talk in front of a group |  |  | 0.758 |
| I get embarrassed very easily |  |  | 0.706 |
| It is important for me to look good | 0.768 |  |  |
| I am in touch with my feelings |  | 0.668 |  |
| I spend a lot of time on my looks | 0.697 |  |  |
| I try to understand what my feelings mean |  | 0.781 |  |
| It takes me time to get over shyness in a new place |  |  | 0.645 |
| I care about how I look in pictures | 0.547 |  |  |
| I care about the way other people think I look | 0.629 |  |  |
| I can always tell when feelings are changing |  | 0.513 |  |
| I like to understand why I do things |  | 0.548 |  |
| I feel scared when I meet someone new |  |  | 0.669 |
| I’m worried about how I do things |  |  | 0.452 |
| I often ask other people how I look | 0.532 |  |  |
| I pay attention to what is ‘in style’ | 0.481 |  |  |
| I am interested in my thoughts |  | 0.555 |  |
| I don't like performing in front of other people |  |  | 0.611 |
| I pick my clothes out carefully | 0.492 |  |  |
| One of last things I do before I leave the house is look in the mirror | 0.571 |  |  |
| I know right away when I am feeling happy or sad |  | 0.352 |  |
| I’m always trying to understand myself |  | 0.735 |  |
| I make sure I look right before I leave the house | 0.595 |  |  |
| I usually know how I feel about things |  | 0.441 |  |
